# Drawing mitochondrial genomes with circularMT

**DOI:** 10.1093/bioinformatics/btae450

**Published:** 2024-07-13

**Authors:** Simon J Goodman, Ian M Carr

**Affiliations:** School of Biology, Faculty of Biological Science, University of Leeds, Leeds LS2 9JT, United Kingdom; Leeds Institute of Medical Research at St James's, School of Medicine, University of Leeds, Leeds LS9 7TF, United Kingdom

## Abstract

**Summary:**

Mitochondrial DNA sequences are used extensively in phylogeographic and phylogenetic studies for a wide range of organisms. With the advent of low-cost, high-throughput “next generation” DNA sequencing, and user-friendly bioinformatics pipelines for generating and annotating whole mitochondrial genome assemblies, the analysis of whole mitochondrial genomes has become an important component of phylogenomic studies for taxa with high species diversity but limited coverage for other genomic resources. An important step in characterizing *de novo* mitochondrial genome assemblies is to evaluate and describe structural rearrangements relative to reference taxa. Accessible tools are needed to help visualize gene and non-coding feature complement, their order, and strand orientation. However, there are few dedicated applications that generate high-quality genome diagrams. Here we present circularMT and circularMT-console that allow users to create highly customizable, publication-quality images, of linear and circular mitochondrial genome maps, either individually or integrated into an analysis pipeline.

**Availability and implementation:**

Both applications are implemented in C#, with binaries, source code, and user guides available on GitHub (https://github.com/msjimc/circularMT). An archive of the published version is available on Zenodo (https://zenodo.org/records/10912319).

## 1 Introduction

Mitochondria derive from the assimilation of symbiotic bacteria during the early evolution of eukaryotes (Sagan 1967) and are present in the majority of eukaryotes as the main site for aerobic metabolism (Voet *et al.* 2006). Although, for multicellular organisms, many of the genes present in the ancestral mitochondrion have transferred to the nuclear genome, mitochondria still possess a small circular genome encoding transcribed sequences, which in animals ranges from 11 to 28  kb (Andersson *et al.* 2003, Kolesnikov and Gerasimov 2012). With few exceptions, animal mitochondria have 37 transcribed sequences, comprising 13 protein-coding genes, 22 tRNAs, and 2 mitochondrion-specific rRNAs, although the order and orientation of features can vary.

In higher organisms, mitochondrial genomes are typically only inherited through the maternal line and so, from a population genetic perspective, behave as a single haploid locus in linkage disequilibrium, without opportunity for recombination (Wei and Chinnery 2020). The transcribed and non-transcribed regions of the mitochondrial genome experience different levels of purifying selection, and therefore have varying substitution rates, creating both highly conserved and hypervariable sequence regions. Metabolically active cells may have several hundred mitochondria, each containing 2–10 copies of their genome. As a consequence, the mitochondrial genome copy number exceeds that of nuclear DNA (Robin and Wong 1988), making them easier to study than nuclear loci, especially when dealing with potentially degraded material, e.g. environmental samples.

These properties of mitochondrial genomes have long made them popular choices as a source of markers for phylogeographic and phylogenetic studies. With the advent of low-cost, high-throughput massively parallel DNA sequencing, and user-friendly bioinformatics pipelines for generating and annotating whole mitochondrial genome assemblies (e.g. Bernt *et al.* 2013, Jin *et al.* 2020), their analysis has become an important component of phylogenomic studies (Tang *et al.* 2014). This is particularly relevant for the study of taxa such as invertebrates that have high species diversity, but are sparsely covered by other genomic resources (Tang *et al.* 2019, Jasso-Martínez *et al.* 2022). As a result, the comparison of mitochondrial genomes is an increasingly important component of biodiversity discovery, taxonomic characterization, and monitoring workflows (Crampton-Platt *et al.* 2015).

An important step in characterizing *de novo* mitochondrial genome assemblies is to evaluate and describe any structural rearrangements relative to reference taxa. Accessible tools are needed to help visualize a mitochondrion’s gene and non-coding feature complement, their order, and strand orientation, through circular or linear schematic plots of the genome. Options for generating such plots include proprietary software such as Geneious Prime (https://www.geneious.com/features/), or open-source resources including the OGDRAW webserver (Lohse *et al.* 2013), or various implementations of the CIRCOS package (Krzywinski *et al.* 2009). However, there are obstacles to their use. For instance, Geneious Prime is a commercially available software application that is able to perform a wide range of sequence analysis and visualization processes, of which the production of mitochondrial genome maps is a small part. As a consequence, the required licensing fee may make the application too expensive for the infrequent generation of mitochondrial genome images. Conversely, OGDRAW is a simple, free-to-use web application that is dedicated to the production of organelle genome images; however, it is limited to the processing of GenBank (Sayers *et al.* 2022)and EMBL-EBI annotation files (Cantelli *et al.* 2022) and offers few options for the modification of the image, with the user encouraged to save the image as postscript file that can be further edited in a dedicated graphics application such as CorelDraw (https://www.coreldraw.com/en/) or Inkscape (https://inkscape.org/). Finally, CIRCOS is a fully featured application, initially created as a Perl module, for the visualization of genomic data, typically in a circular fashion. While CIRCOS is very flexible and feature-rich, it requires a level of programming/command line expertise that many biological researchers do not possess and so its use may present a daunting learning curve to infrequent users. An implementation of CIROS hosted on Galaxy (The Galaxy Community 2022) has resolved some of these issues, but its use can be slow due to the multi-user nature of Galaxy, and currently has limited documentation.

Here we introduce circularMT, a desktop and command line application for producing high-quality circular and linear maps of mitochondrial genomes from a wide range of input file formats. While simple to use, the application allows the user to modify the genomic maps, such as correcting erroneous annotation pipeline artifacts (e.g. non-coding features assigned with low confidence), and a high level of customization for the graphical presentation of features of interest.

## 2 Design and implementation

The object-orientated language C# was used to create the desktop application: circularMT and the command line program: circularMT-console. While primarily a Windows-based program, they can be run on several POSIX-compliant operating systems, such as Linux, macOS, and BSD using Wine (https://www.winehq.org/) as described in the user guide. The primary interface of circularMT consists of a single window containing controls on the righthand panel that modifies the displayed genome, drawn on the plot area to the left, either directly or via a series of process-specific dialogue boxes; whereas circularMT-console is a command-line-driven implementation that can be used for batch processing files or as part of an analysis pipeline.

To aid useability, both programs are able to process a wide range of file formats most notably GenBank annotation files and those exported by MITOS (Bernt *et al.* 2013), a widely used organelle annotation web application currently hosted on Galaxy. While these files, and others like GTF and GFF formatted files, have a rigid structure, other file types are more loosely defined and must conform to the requirements outlined in the user guides.

**Figure 1.**
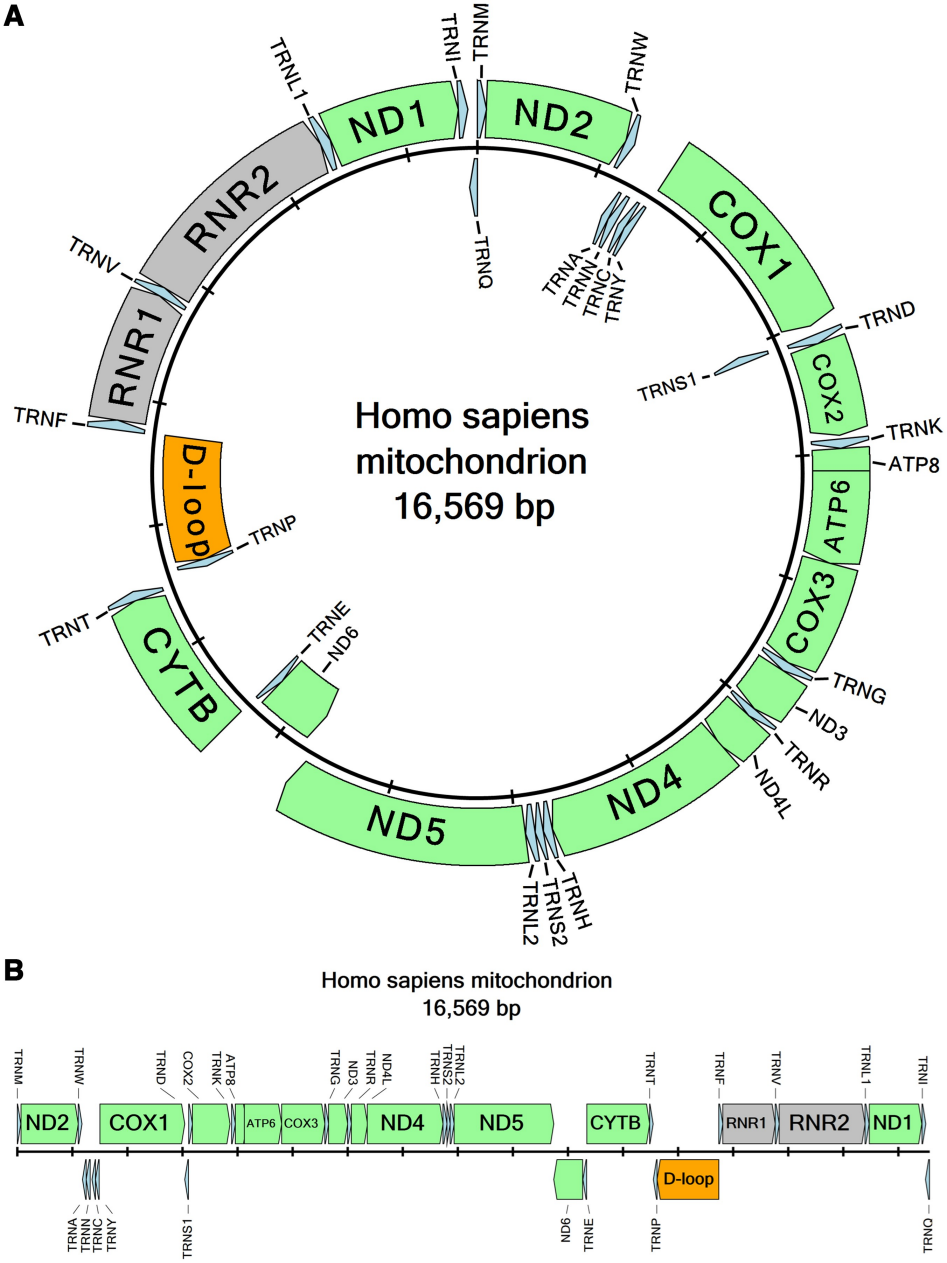
The human mitochondrial genome (NC_012920.1) draw as: (A) circular and (B) linear maps by circularMT using the sequence’s GenBank annotation file.

As circularMT parses the input file, it determines each feature’s genomic coordinates and orientation that are required to draw the map, along with the optional data of the feature’s type (i.e. tRNA, rRNA, or protein-coding gene), and any names it has been given. This information is then used to create either a linear or circular map of the genome in which features are represented as arrows (pointing 5′–3′). For circular maps, the arrows form two rings with the outer circle representing the forward strand and the inner circle the reverse strand, while linear maps are arranged as two rows of arrows with the upper and lower rows representing the forward and reverse strands, respectively (Fig. 1).

Where possible, feature names are drawn within the arrow, but if the text is too long it is written by the side of and at 90° to the arrow. tRNAs often form tandem arrays of up to five genes, which due to their short sequence length may result in externally drawn text clashing, consequently, circularMT will automatically modify the text’s position to limit this overlap, while also offering a method for the user to manually adjust their position. For circular diagrams, the map will resize itself such that the names of features do not extend beyond the edge of the image. It also allows the user to manually modify other aspects of the map; for instance, the user can add, delete, and rename features to correct errors in the annotation. It is also possible to customize the color scheme of one or all the features of a specific feature type to produce a more informative color-coded image to meet the user’s requirements.

The *de novo* assembly of mitochondrial genomes results in contigs in both orientations that start at random points in their genome. Consequently, circularMT allows the user to switch the annotation’s strand orientation and change its start point such that a set of images conform to a standardized style; for instance, all maps start at the beginning of the methionine encoding tRNA gene, which is placed on the forward strand.

The command line application circularMT-console is intended for the batch creation of images from multiple annotation files, or to be integrated into an analysis pipeline. As it runs in an unsupervised manner, it has few user-set parameters and is primarily intended to create a first-pass image for use in an analysis pipeline report, with circularMT used to create bespoke maps used in a more formal setting.

Once a genomic map has been produced by circularMT, it can be saved as one of several image formats (TIFF, JPEG, PNG, or BMP) at a resolution between 100 and 1000 dpi allowing the images to be used in publications, theses, or reports (see Fig. 1). Images created by circularMT-console are saved as 300 dpi, TIFF, JPEG, PNG, or BMP files.

## Data Availability

No new data were generated or analysed in support of this research.
